# Identifying the processes of change and engagement from using a social network intervention for people with long‐term conditions. A qualitative study

**DOI:** 10.1111/hex.12839

**Published:** 2018-10-14

**Authors:** Ivaylo Vassilev, Anne Rogers, Anne Kennedy, Chad Oatley, Elizabeth James

**Affiliations:** ^1^ NIHR CLAHRC Wessex University of Southampton Southampton UK; ^2^ School for Sport, Health and Social Sciences Solent University Southampton UK

**Keywords:** behaviour change, intervention, practices, self‐management, social networks

## Abstract

**Background:**

Personal and community networks are recognized as influencing and shaping self‐management activities and practices. An acceptable intervention which facilitates self‐management by mobilizing network support and improves network engagement has a positive impact on health and quality of life. This study aims to identify the processes through which such changes and engagement take place.

**Methods:**

The study was conducted in the south of England in 2016‐2017 and adopted a longitudinal case study of networks design. Purposive sample of respondents with long‐term conditions (n = 15) was recruited from local groups. Barriers and facilitators to implementation were explored in interviews with key stakeholders (5).

**Results:**

Intervention engagement leads to a deepening of relationships within networks, adding new links and achieving personal objectives relevant for improving the health and well‐being of users and network members. Such changes are supported through two pathways: the mobilization of network capabilities and by acting as a nudge. The first is a gradual process where potentially relevant changes are further contemplated by forefronting immediate concerns and negotiating acceptable means for achieving change, prioritizing objective over subjective valuations of support provided by network members and rehearsing justifications for keeping the status quo or adopting change. The second pathway changes are enacted through the availability of a potential fit between individual, network and environmental conditions of readiness.

**Conclusions:**

The two pathways of network mobilization identified in this study illuminate the individual, network and environmental level processes involved in moving from cognitive engagement with the intervention to adopting changes in existing practice.

## 1 BACKGROUND

There is a recognition that providing person‐centred care and understanding what people with long‐term conditions value in relation to self‐management requires exploring the contexts and ways in which social ties and resources shape everyday interactions and mechanisms through which changes in existing practice are negotiated.^1^, ^2^ Social network interventions designed to mobilize resources have to compete alongside pre‐existing practices and manage interactions between people and their contexts to ensure the acceptability, workability and integration of new ways of doing things in everyday life.^3^, ^4^

Two ideas underlie the development, deployment and successful implementation of a social network intervention Genie (generating engagement in network involvement). Firstly, self‐directed support for managing health can be accessed through people's social networks and engagement and is predicated on the wide range of connections available to people in open settings (family, friends, groups, acquaintances and pets). The latter provide opportunities for connectivity reciprocity and accessing resources amongst network members for support.^5^ In terms of living and managing well with a long‐term condition (LTC) this means realizing and sustaining valued activities and participating in social, cultural and group activities 6, 7 and maintaining and developing valued reciprocal relationships with others within proximate communities.^8^, ^9^

The social network intervention considered here is facilitated and includes mapping and reflecting on the composition of personal networks, eliciting preferences, and considering options for engaging with local and online resources, groups, people and organizations.^4^ It is predicated on the notion that people with long‐term conditions are more likely to engage with relationships, things and activities they choose and value.^8^ When delivered by trained facilitators in a community setting (supporting people with diabetes and early stage CKD), Genie led to an increase in diversity of participants’ networks, greater engagement with community activities and had a positive impact on blood pressure, health‐related quality of life and lower health‐care utilization.^4^, ^10^ However, uncertainty remains about the processes through which these changes occur and how network engagement activated by the intervention interacts with the relevant contextual, network and individual level factors within people's everyday lives. Here, we are interested in developing a better understanding of how this process is shaped by the structure of people's networks and the immediate environments within which they are located.

## 2 METHODOLOGY

### 2.1 Recruitment and data collection

The study was conducted in the south of England in 2016‐2017 and adopted a longitudinal case study of networks recruiting a purposive sample of respondents who were over 18 years old and living with long‐term conditions (n = 15). Local voluntary and community groups that supported this population were visited in person by a researcher or a PPI representative or were contacted via online support networks. Respondents included people of different ages (45‐84), and varied by gender, income, employment and marital status, and number of network members (Table 1).

**Table 1:** Participant characteristics

ID	Gender	Age	Marital status	Health conditions	Years in f/t education	Highest level of education	Most recent occupation	Employment	Income	Number of network members
1	F	50	Married/ive‐in partner	Behcet's disease, associated arthritis	13	College	Clerical/admin/receptionist	Part‐time	Lower than average	15
2	M	70	Married	Parkinson's, epilepsy, arthritis, depression, intestinal disorder	13	College	Woodwork teacher	Retired	Lower than average	13
3	F	83	Married/live‐in partner	Heart disease	11	Secondary	Teacher	Retired	Blank	14
4	F	48	Married/live‐in partner	Multiple sclerosis	18	University	Resident involvement officer	Full‐time	Higher than average	14
5	M	84	Married	Parkinson's, arthritis	19	University	Town planner	Retired	Higher than average	11
6	F	45	Live‐in partner	Multiple sclerosis	13	College	Personal Assistant	Unable to work due to LT ill health	Lower than average	8
7	M	69	Married/live‐in partner	Heart disease, in recovery from prostate cancer	16	University	Fire Officer	Retired	Higher than average	18
8	M	68	Married/live‐in partner	Heart disease, arthritis, nerve damage	12	University	Prison officer	Retired	Lower than average	9
9	M	81	Married/live‐in partner	Heart disease, arthritis, diabetes T2	13	Secondary	Maintenance manager	Retired	Lower than average	15
10	M	45	Single/never married	Multiple sclerosis	12	College	Painter	Unable to work due to LT ill health	Lower than average	13
11	M	69	Widowed	Atrial fibrillation, arthritis	11	Secondary	Van driver	Retired	Lower than average	15
12	M	81	Widowed	Heart disease, arthritis, prostate cancer	10	Secondary	Mechanic	Retired	About average	19
13	F	56	Divorced	Arthritis, depression	12	Secondary	Carer	Unable to work due to LT ill health	Lower than average	10
14	F	45	Single/never married	Arthritis, asthma, anxiety/depression, plantar fasciitis	11	Secondary	Carer	Working part‐time	Lower than average	16
15	F	66	Widowed	Castleman's disease, diabetes, heart disease, arthritis	10	College	Carer	Retired	Lower than average	10

Each participant met with a facilitator face‐to‐face at two time points, with a 3‐month interval in‐between. The baseline meeting lasted 45‐90 minutes and was followed by a qualitative interview with a researcher, lasting approximately 60 minutes. The 3 months follow‐up focused on the network mapping stage and lasted 30‐40 minutes. Facilitators came from a range of backgrounds including care navigators, community navigator, local area co‐ordinator, PPI representative, public health practitioner and applied health researchers.

We collected qualitative data about the processes of implementation and the outcomes of engagement or non‐engagement with personal networks and online and off‐line resources. We used observation and in‐depth interviews at two points in order to elucidate the complexities of social practice and multiple actors over time.^11^ A researcher observed intervention delivery using note‐taking and video recording, and focused on user‐facilitator interaction, and contextual, individual and network factors of potential relevance for users in adopting changes in practice. Following each observed case study, the researcher interviewed the participant and wrote field notes including impressions of how the intervention was used and accepted. Three months after the intervention all respondents were interviewed again in order to explore changes in the structure of personal networks, engagement with social network support, and accessing services and devices relevant for self‐management support. The follow‐up interviews included a “think aloud” method 12 where the interviewees were asked to comment on the challenges they experienced in using the resources discussed at baseline. We were interested in how users approached, accessed, navigated and engaged networks and resources of support as informed by previous evaluations of e‐health and SMS tools.^13^, ^14^

In order to explore how the social and physical environments shaped network activation, practice change, and to identify barriers and facilitators to the implementation and long‐term sustainability, we set up a working group, which included health trainers, representatives of adult services, public health, representatives of voluntary and community organizations (n = 15). We kept extensive notes of working group meetings and informal discussions with key local decision makers and interviewed five members of the WG involved with different aspects of the implementation process (managers and intervention facilitators from voluntary organizations and local service providers).

### 2.2 Data analysis

The analysis drew on normalization process theory and focused on understanding how coherence and cognitive engagement developed during the intervention 4 led to engaging users and network members in adopting changes in their everyday practice and the reflexive monitoring of this process over time.^15^ A coding and analysis framework described the extent and nature of changes made by users over three months; the relevant factors, the types of work done by network members, and the processes involved in making these changes; the selective engagement of network members (navigation) and the process of reshaping existing relationships (negotiation) in making new connections, improving capacity to enact healthy behaviours, improving well‐being, reducing isolation.^16^ The coding framework was agreed collaboratively by members of the research team. Any coding differences were discussed at regular meetings in order to reach agreement. In analysing the data, we used comparisons and drew out new improvements and benefits specific to individual circumstances.

## 3 FINDINGS

Our findings related to processes and change in personal networks. Most users reported increased number and frequency of network contact identifying additional members of personal communities who they thought were important to them, but who had not been previously identified (Table 2). The intervention was effective in extending user networks by adding new groups and activities (eg walking group and Parkinson's support), tools (eg pedometer, weight‐watcher points converter, laptop and mobility scooter) and engagement online (Table 3). Users with small and family or friend‐centred networks 17 reported most change both in engagement with and extending personal communities. Participants with diverse networks (who also had the highest socio‐economic status) reported smaller number of changes. The process of engaging with networks towards changing existing practice is illuminated in three themes: building capacity for articulating, reframing and re‐orientating relationships and capabilities; nudging a link to enabling environments and activated networks; and environmental fragilities in engaging and sustaining practice change.

**Table 2:** Network changes at time 2^a^

ID	Extending networks			Network engagement		Changes within networks
	New groups or activities added	New things added	Online	Reflection on existing support	Increased contact with existing groups	
1	a	a	a	a	a	5/5
2		a		a	a	3/5
3				a		1/5
4				a	a	2/5
5				a		1/5
6	a		a	a	a	4/5
7	a					1/5
8					a	1/5
9				a		1/5
10		a	a	a	a	4/5
11				a	a	2/5
12	a	a		a	a	4/5
13	a	a				2/5
14			a	a		2/5
15	a	a	a	a	a	5/5
Outcome changes	6/15	6/15	5/15	12/15	9/15	

a Time 2 refers to changes 3 months after the intervention (time 1).

**Table 3:** Extending networks at time 2

ID	New groups or activities						New things					Online				New and activated relationships and support								
	Walking	Dog walking	Gym and Aquafit	Hair beauty salon	Support or activity group	Yacht club	Mobility scooter	Pedometer	Vouchers	WW points converter calculator	Lift in block	Hyster sisters	Facebook	Online activities	Parkinson's nurse	Relatives	Hairdresser	Friends	Personal assistant	Care staff at residential home	Refugee worker	Specialist	Cleaner	Housing scheme manager
1	*							*																
2									*						*									
3																								
4																*								
5																								
6				*										*			*	*	*	*	*			
7						*																*		
8																*		*						
9																								
10							*									*								
11					*																			
12																*							*	
13	*										*	*							*					*
14		*											*											
15	*		*							*														

### 3.1 Building capacity for articulating, reframing and re‐orientating relationships and capabilities

Respondents found that visually mapping their network and discussing this with the facilitator opened up space where they felt listened to, “had the opportunity to express feelings,” it was like “a warm comforting exercise” (ID7) that allowed “time for myself” (ID1). It was apparent that discussion opportunities where one did not feel “categorised, stigmatised” were valued by respondents but not always available.

…over the last fortnight. I seem to have developed a better attitude towards things. I don't know how, but it's probably talking to you two outside of my normal circle. […] What is this space about? friends who dealt with my emotional needs, you don't deal with my physical needs, but somehow you dealt with my mental needs. I'm more willing to be a bit more pro‐active than I was. It's all just starting …but it takes time. (ID6)

This assessment was also reflected in accounts of facilitators who thought it addressed an existing gap in their practice.

…it actually starts a conversation […] it breaks the barriers if somebody is shy or doesn't like talking to people […] Because we listened, we got to know the person, we thought about the whole person not just if they come to us because they want to lose weight but actually there's no point in talking to someone about losing weight if their home life is not good, they've got no money. […] It's really building up the picture of that person's life and how their circumstances are, and in a way Genie goes from one to another so it's quite nice because you can move on without actually asking too many questions.’ (SH1)

The discussion at T1 and T2 made it apparent that in some cases there was lack of fit between opportunities for engagement, network capacity and personal priorities. Using Genie supported a process of articulating and engaging with personally defined objectives and personal community members. Cognitive engagement offered a set of reference points for reframing self‐management support in network terms and for identifying potentially relevant changes to existing practices. However, these needed further thinking through in terms of identifying the rationale for making changes and identifying alternative activities that might lead to more substantive change. This process included negotiating objectives and engagement with network members, forefronting the items of most preference and value and rehearsing justifications for these.

#### 3.1.1 Negotiating objectives and engagement with network members

The main initial focus was on engaging network members and aligning users to local preferred activities which the participant had not previously tried. However, the option of immediate engagement was not always possible if the options were seen to be currently unachievable due to incapacity, or required yet to be negotiated access, resources time and effort.

Yes, I still want to join the W.I. which is one of the things that I want to do but it will be a few weeks until I feel well enough to walk up there because that's the only thing, I've got to get myself able to walk longer distances. The same reasons as well there is an art club which was mentioned […] that is my arthritis and it would be too expensive to go back and forth on the bus […] I was hoping possibly if I made contact with people that were within these clubs there may well then be somebody that lives locally to me or comes by this way that I could cheekily cadge a lift off. (ID13)

Being able to engage, network members opened up possibilities for some to start addressing personal objectives to integrate network members with making a positive stepped change. Thus, while one of our male respondents (ID10) recognized the value of going to the gym his main priority was expressed as “building up strength.” He had been able to achieve that by working at home and “getting his room done” with the help of his former work colleague and friend. As a result of managing to increase his capacity to work (from two to five hours a day), he was at T2 looking forward to extending his physical activities. This included beginning work in his garage “getting the furniture repaired when it is not that cold” and painting the house before getting back in contact with the Genie facilitator in order to start going to the gym.

The ability to mobilize support was shaped by considerations of what each respondent thought was acceptable in balancing individual and network responsibilities while trying to achieve ends of mutual value. A female respondent (ID1) identified losing weight as one of her objectives, and during the Genie discussion, her partner appeared as a potentially key point of support due to his extensive knowledge about diet and cycling. Although the respondent did not doubt the availability of such support, she was wary about drawing on it. She thought that her partner would take over and that his approach did not suit her: “You are in it to win it with [partner]…. My God, yes, he would have all the food out the house… I would shift a stone in about three weeks if [partner] was in charge.” She felt this would make the relationship unequal and an obstacle to finding things that she and her partner could enjoy doing together as a couple.

Engagement also had a direct impact on the activities prioritized by other network members. For example, the wife of another participant, who was present during the intervention, found that Genie made her reflect on her own network, leading to drastically reducing her working hours, opening “a lot more free time” and joining a women carers forum which she now attends once a month. She also got interested in visiting one of the community centres that was identified as potentially relevant for her husband as she wanted to make sure this might be appropriate for him while also extending her own network:

…apparently, they care for carers as well, so after Christmas maybe when I settle down a bit I might be able to go over there and then if I can go over and join groups there then perhaps [husband's name] can come with me and then I'll be there to deal with any problems. (ID2)

For some users, engagement with the intervention failed to deepen or extend network engagement, but brought about an enhanced awareness of the value they put on maintaining existing activities and the individual and network resources that these required (ID3).

#### 3.1.2 Forefronting evaluations of network support

The work that different network members do to manage things and the value of this to respondents was sometimes presented in procedural terms with clearly defined boundaries and responsibilities.

However, in some instances respondents started identifying potential tensions between subjective and objective valuation.

They'd better go in with everybody else if you've got room…there's probably someone really important I've forgotten…Daren't leave any of the children out or we'll get into trouble. (ID3)

This process of reflection tended to lead to asking concrete questions about value, responsibility and contribution. Thus, discussions at T2 led to the emergence of a stronger emphasis on the objective contributions made by network members rather than on subjective value and the normative expectations associated with specific ties. Respondents sometimes found it difficult to acknowledge the limited role that family members played in supporting them and were reticent in physically moving them to the outer circle of the network diagram. However, in some cases they were able to articulate a shift towards prioritizing seemingly objective valuations. For example, at T1, ID12, whose support network was fairly limited with most regular face‐to‐face contact coming from neighbours, acquaintances and health and social care support, discussed his unhappiness with his estranged relationship with his daughters. He talked about changing his will to reflect the loss of relationship, which troubled him as they were “his blood” yet “they weren't interested.” He contrasted that with the supportive relationship with his son‐in‐law and stepbrother who, even though living in the United States, came over and stayed with him when his wife died. At T2, the respondent put many members of his US family on the diagram and had regular FaceTime conversations with them.

Recognizing the value of some of the less intimate (weak) ties was in some instances subsequently accompanied by the extending and deepening of such relationships. Thus, ID6 thought her volunteering work “is a lifeline” that offered her a respite from the difficult relationship with her partner, and at T2, she was able to increase the time spent there. Another respondent (ID11) felt that he improved his skills and deepened his involvement with the walking group he was attending when he started playing the guitar with one of the group members.

#### 3.1.3 Rehearsing justifications in engaging others

Renegotiating relationships and roles, and mobilizing network engagement involved developing justifications for change to support arrangements that were acceptable for respondents and appeared so for members of their personal community. The initial Genie discussion led to revising and rehearsing changes to views and positions about individuals within their networks. For example, although ID10, who had MS, experienced financial difficulties he found it difficult to accept that he might need to apply for carer's allowance to which he was entitled. He felt that this was morally wrong, a view shared by his mother as she thought “he gets enough already” and did not need to accept additional financial support. Although shifting this view was difficult for the respondent, he resolved it by arguing that the money would be spent on getting “nice things” for his parents and going on holidays “to the cottage in Cornwall” that “we all love.” Additionally, this was justified because his mother was doing “huge amount of voluntary work for other people and deserves some acknowledgement.” But this money would also make it possible to help financially his partner and stepdaughter in Argentina. At T2, he took a decision to ask the Genie facilitator to help him with “doing the forms” and claiming the allowance.

### 3.2 Nudging a link to enabling environments and activated networks

For some participants, the intervention coincided with the contingencies of a fortuitous combination of an activated personal community and a supportive environment. In this context, the intervention acted as a tipping point towards changes that were already part of an ongoing discussion within people's personal communities. For example, the wife and daughter of one respondent were in the process of looking for someone to help him get up and dressed in the morning, as his wife was finding it increasingly difficult to help him physically. The respondent was concerned how he would cope as “I wake at different times” and that if he got different carers he would “have to teach them my routines” although he recognized that “…my daughter is anxious that I shouldn't wear my wife out” (ID5).

At T2, the personal care has been arranged, fitting in with a neighbour who had the same carer so that “we would probably fit in around her. So, if she is seen say at 9 am, she'd come here at 9.30…probably once a week.” Although the respondent still felt “a bit ambivalent because I've never had that kind of support before,” he and the members of his family were able to make this change more acceptable by likening it to them employing a weekly cleaner who has now “become more like a friend” and “a ray of sunshine.”

In other cases, participating in the intervention created a “nudge”^18^, ^19^ towards engaging with resources and opportunities available in users’ environments. During the intervention at T1, ID1 identified joining a walking group as one of her objectives, but could not link up with the option Genie provided as it did not fit with her timetable. At T2, she added a pedometer in her inner circle. The pedometer was made available to her for free at work, so that “you just had to go through occupational health, you could do it if you want just through like a bit of a fitness thing really,” and its use was also sustained by the supportive environment, the involvement of her colleagues, and because one “can see what others in our area are doing […] you can see how you are in the table.”

This respondent was able to extend her walking activity by arranging to walk with her daughter “two or three nights a week” and by linking up with her friend with who she used to walk in the past. In explaining this change, a narrative link to other contextual and personal factors was made: “had my knee done,” “got over the op and had the stitches out,” and the “summer came and the lighter evenings came and we went out to different things,” “different garden centres on the island.” Similarly, a nudge might be made towards reorganizing network support in a new context. For example, one participant, with multiple mental and physical health problems who lived alone, realized she was quite isolated and that most of her contacts were online or by phone. The discussion at T1 “made me think about looking at things out in the wide world to do and not, because I can be quite self‐insulated because of the things I'm interested in.” However, there were barriers to enacting the changes identified as important until she moved to new housing. Her previous accommodation was difficult to access “I was living in a flat that was like 60 odd stairs up to my front door and I was unable to access outside very well,” which together with her high levels of anxiety compounded the feeling of physical and social isolation. Since moving to the new flat she has been able to reorganize her network and engage the support of people who she met recently.

[…] Since living here I have found that if there are days when I just think I could do with a chat or I feel a bit isolated then I just pop down in the lift and if [warden] is around or there might be somebody in the laundry room you can have a chat to, or the communal area, or just go for a walk down to the shops. There are people around here, and like I said I'm quite friendly with [neighbor]

This enabled the respondent to undertake longer walks made easier by the new support and availability of a lift to get downstairs and provided a cognitive link to the adoption of a new medication regimen. So, she is walking more in part “because I'm worried […] because they put me on that Clexane to prevent thrombosis and DVT and so obviously I need to be mobile.”

For all respondents, engagement with new activities tended to fit with familiar activities, such as joining walking groups or starting walks with a network member, while more complex and unfamiliar changes were less likely to materialize as they required more time for engagement and additional support from members of their network.

### 3.3 Environmental fragilities in engaging and sustaining practice change

Participant engagement with the intervention highlighted differences in how sustainable engagement with new activities was co‐shaped by the type of groups accessed, the availability of longer‐term facilitator support and the structure of personal communities. Some of the organizations that users linked with had existed for a long time, had stable funding structure and opened possibilities for user engagement that were self‐organizing and entirely focused on the evolving user preferences and needs.

Oh yes, this last couple of weeks there is this one guy who has a massive allotment and he's been bringing runner beans, tomatoes and loads of veg and he puts them there and you take what you want and just make donation to the club. Things like that which is nice. You are building up a social group, aren't you and the of course there are the activities they put on, trips out, there is a variety she on, they have quizzes, …I put quizzes, together, this is something I enjoy doing…We are trying to start up a pétanque club. (ID8)

Such organic growth and engagement with network members although narrower in scope was discussed in relation to engagement with self‐organizing groups of colleagues, the “banter club,” or church groups (ID5).

However, other groups were small, poorly funded and their continued existence depended on the ongoing support of the users. ID11, for example, relies heavily on support from a staff member at one of the resource centres he attends. They have been sorting a lot of household/domestic issues together, and at T2, the respondent was using “we” rather than “I” to denote a feeling of support.

Well, the fridge is leaking and we might have to get another fridge because there's all water coming out of it. It's not running now at the moment but I think it's on its way out’…also […] [staff member] is keeping an eye on with the money just to make sure we're not going too low’.

However, when he started going to a second resource centre, he did not want to appear to favour one centre over the other. “I just think, well, I can't let [group lead] down because I don't want to give the walking up because that is good for me and [she] said I understand if you go out with [other group].” So now he goes to each centre on alternate weeks. For this respondent, the engagement with the two groups required deepening relationships leading to high levels of personal responsibility towards group members to allow continued engagement.

Similarly, ID14 relied heavily on the Genie facilitator when attending new activities and engaging socially. As this support was not available long term, her new links appeared fragile.

there might be some things I can do, get involved in. The thing is, because I get anxiety as well, sometimes I won't, I think oh yes, I want to do it, and then I won't. Like when I first went to do the cooking, [facilitator] wanted me to do it, and he said he'd meet me down there, and that morning my anxiety kicked in, I felt sick, I had an upset stomach, I felt really ill. But I made myself go, because I knew [he] would be waiting for me outside. So, I made myself go because I didn't want to let him down. (ID14)

Linking users with small more fragile organizations brings with it support which is less likely to be sustainable over time and also creates new relational work for users which might also be unsustainable.

The only changes really are [facilitator] is no longer working with me because her job remit they changed what they were now doing so I'm not in touch with her anymore, and [another link worker] has changed down from weekly to monthly now, so a little less support from her which eventually will be phased out completely I think. (ID13)

Uncertainty about the remit of services, roles and responsibilities of link workers, and long‐term funding commitment were also recognized by the local stakeholders as having an impact on the implementation of Genie and on maximizing its effectiveness in supporting network activation and change.

[people with complex circumstances] need more support than just identifying there is a group at the end of the road, they might not actually be able to get to the end of the road so they need more support with finding a volunteer who can potentially pick them up for example to take them to that group. (SH2)

This also reflected a broader systemic problem:

…engagement I think, wider than just health and social care would be great because ultimately if we are looking at things holistically that would be great and I think at times it's been very health and social care orientated as most things on the Island tend to be. I think that would help to support it and getting that wider network. Again, in a wider system barriers which is hard for Genie to be able to get over that because actually the system needs to sort itself out first … (SH5)

The difficulties in achieving the necessary systemic consolidation were illustrated by the tension in stakeholder accounts between, viewing Genie as potentially useful for a broad set of users with a wide range of needs and circumstances, while, emphasizing the need to identify where and for who it can work best and how its impact could be assessed in relation to key performance indicators.^20^ The uncertainty about division of responsibilities, long‐term commitment and funding, data storage and security (SH4) affected engagement and enthusiasm about Genie and made it difficult to work towards operationalizing, embedding and sustain its use over time (SH1). Within this context, most stakeholders thought that the sustainability of the intervention might benefit from taking a complementary top‐down approach, “the strategic buy in as well” (SH5), with a clear set of commitments, a “system ownership” so that Genie forms part of mainstream formalized work streams that has gravitas, for example reporting to Joint Commissioning Board of the Health and Wellbeing Board (SH3).

## 4 DISCUSSION

This study through illuminating underlying mechanisms contributes new insights relevant to theories of readiness to change and interventions which include a social environmental dimension to self‐management support through providing socially based options to improve health and well‐being (eg social prescribing and asset‐based approaches).^21^, ^22^ Identifying rationales for making changes through engaging with options that might lead to change resonates with behaviour change and self‐management theory which highlight the need for building a relationship of trust through *rapport*, establishing in people's minds a need to be engaged in new practices and finding workable *solutions* that are most likely to be adopted by individual patients (eg Transtheoretical Model of Change, Motivational Interviewing, Motivational Model of Patient Self‐Management and Patient Self‐Management).^23^, ^24^, ^25^, ^26^

Engagement with a social network intervention leads to deepening of relationships within personal communities of support, extending networks by adding new links and activities and achieving personal objectives relevant for managing the health and well‐being of users and members of their networks. This study extends our understanding of the processes through which such changes take place.^4^ The findings indicate that cognitive engagement leads to the mobilization and development of network capabilities and can act as a nudge towards the realignment of resources and support.

Making changes to existing practices through the mobilization of network capabilities involves a number of processes. These are forefronting the immediate concerns of users and members of their personal communities, negotiating and activating the possible means for achieving these. It may include prioritizing objective over subjective valuations of the support provided by network members and rehearsing justifications for keeping the status quo or for adopting change. The Genie intervention helps by identifying possible activities that are dormant in a person's life or novel (eg join an art group and reconnect with friends). These possibilities act as a set of reference points for further thought and articulation in relation to the consideration of personal capacity, immediate priorities (eg get physically fitter) and contextual factors. This requires additional relational work (ie efforts to negotiate mutually acceptable changes in relationships with others and/or selectively navigating out of situations to avoid the need for renegotiation**)**, which may involve reframing expectations, adopting changes through new justifications, developing narratives and rehearsing the sequencing of potential changes in practice. This is likely to be a gradual and reflexive process. By contrast, the nudges towards realignment of support were seemingly made possible through the availability of a potential fit between individual, network and environmental conditions of readiness. In such cases, engagement with Genie acts as a steer towards readjustment within conditions that already exist and only require minimal change. For example, engagement with weak ties within personal communities could potentially act as a nudge towards change by providing a missing link or type of support that makes everything else fit (eg acting as a companion for walks, where starting walks is already an immediate priority due to professional advice about taking a medication, where there is easy access to a safe and walkable area, and past but discontinuous experience of going for walks).

Our findings suggest that the mobilization of network capabilities might be seen as a useful pathway to supporting changes to individual circumstances because it highlights a process of engagement with the current concerns of individuals and their network members. Navigating and negotiating relations within personal communities is a condition for engagement with network‐based interventions such as the one reported here with indications that it enhances existing capacity for long‐term condition management work. It may also indicate the building of individual and collective resilience and flexibility in adapting to the changing needs of people with LTCs in terms of managing everyday life.^25^, ^26^ In this regard, access to different types of ties which make up a personal community is likely to be relevant through the properties of interaction. Thus, weak ties can act as a counter to strong tie connections by avoiding the need to make changes in relations that are both valued and difficult to change, avoiding or reducing the burden on strong ties, providing a wider range of options.^27^ This study indicates that people with limited resources, smaller networks and lower levels of community connections are more likely to be supported through network engagement and negotiation.

## 5 CONCLUSIONS AND POLICY IMPLICATIONS

The Genie intervention appears to be effective in bridging the gap between cognitive engagement with a network framed understanding of self‐management support through network mapping and preference elicitation, and its activation in the context of people's everyday life. The two pathways of network mobilization towards adopting practice changes identified illuminate interdependencies between individual, network and environmental level processes and highlight potential challenges for its future use as a scalable intervention for supporting long‐term condition management. The impact of Genie in activating networks and supporting behaviour change is likely to be enhanced by the availability of local resources enabling people to live well.^9^

## CONFLICT OF INTEREST

The authors declare that they have no competing interests.
